# Practical Issues in Developing a Culturally Tailored Physical Activity Promotion Program for Chinese and Korean American Midlife Women: A Pilot Study

**DOI:** 10.2196/jmir.6454

**Published:** 2016-11-21

**Authors:** Wonshik Chee, Sangmi Kim, Tsung-Lan Chu, Hsiu-Min Tsai, Xiaopeng Ji, Jingwen Zhang, Eunice Chee, Eun-Ok Im

**Affiliations:** ^1^ School of Nursing Duke University Durham, NC United States; ^2^ School of Nursing University of Pennsylvania Philadelphia, PA United States; ^3^ Department of Nursing Linkou Chang Gung Memorial Hospital Tao-Yuan Taiwan; ^4^ Department of Nursing Chang Gung University of Science and Technology Taoyuan Taiwan; ^5^ Department of Nursing Chang Gung University Taoyuan Taiwan; ^6^ Department of Communication University of California Davis, CA United States

**Keywords:** Web-based intervention, physical activity, midlife, women, Asian American women, Korean American women, Chinese American women

## Abstract

**Background:**

With advances in computer technologies, Web-based interventions are widely accepted and welcomed by health care providers and researchers. Although the benefits of Web-based interventions on physical activity promotion have been documented, the programs have rarely targeted Asian Americans, including Asian American midlife women. Subsequently, culturally competent Web-based physical activity programs for Asian Americans may be necessary.

**Objective:**

The purpose of our study was to explore practical issues in developing and implementing a culturally competent Web-based physical activity promotion program for 2 groups of Asian American women—Chinese American and Korean American midlife women—and to provide implications for future research.

**Methods:**

While conducting the study, the research team members wrote individual memos on issues and their inferences on plausible reasons for the issues. The team had group discussions each week and kept the minutes of the discussions. Then, the memos and minutes were analyzed using a content analysis method.

**Results:**

We identified practical issues in 4 major idea categories: (1) bilingual translators’ language orientations, (2) cultural sensitivity requirement, (3) low response rate, interest, and retention, and (4) issues in implementation logistics.

**Conclusions:**

Based on the issues, we make several suggestions for the use of bilingual translators, motivational strategies, and implementation logistics.

## Introduction

Increasing use of the Internet by racial/ethnic minorities has prompted health researchers to be interested in using the Internet as a method for data collection and as a medium for interventions for racial/ethnic minorities. For example, Asian Americans as a racial/ethnic group use computers more than any other racial/ethnic groups [1-3]. About 66% of Asian Americans reportedly use smartphones [3,4], and about 50% of them were reported to use tablets [5]. Thus, it is natural to assume that a study or an intervention using the Internet would work very well in this specific population.

Web-based interventions, furthermore, are reported to be effective for isolated or marginalized people with stigmatized conditions (eg, human immunodeficiency virus infection, depression) and for underserved people such as racial/ethnic minorities and rural populations [6,7]. The literature even supports that Web-based programs would be more beneficial for racial/ethnic minorities than for white people [6,7]. Indeed, Web-based programs reportedly have great potential to narrow racial/ethnic disparities in health and illness experience and to reduce barriers to care by providing information and support to racial/ethnic minorities [6,7]. Furthermore, socially marginalized or deprived people (eg, those with low income) are reportedly more interested in eHealth than are others, and Internet resources are valued by those who cannot easily establish equal and honest relationships with their health care providers in clinical settings (eg, racial/ethnic minorities) [8-13].

Despite these well-known benefits, racial/ethnic minorities, including Asian Americans, are reported to use Web-based programs at a minimal level [14-22]. As a plausible reason, it has been pointed out that Web-based programs have rarely been tailored to racial/ethnic minorities [14-22]. Thus, researchers have reported the necessity for culturally tailored Web-based programs for racial/ethnic minorities to enhance the appeal and accessibility of the program to these groups [20,21,23-25].

Indeed, few existing Web-based physical activity programs were culturally tailored to Asian Americans despite their great presence on the Internet [6,26-31]. Rather, most of these programs targeted patients with diabetes, adolescents, or general adult populations [16,22,32]. For example, Wanner et al developed and tested a Web-based physical activity intervention for a general online population with positive results [9]. Massoudi et al developed an app for personal health records that delivered a physical activity promotion intervention for sedentary adults, also with positive findings [8]. However, none of these existing programs were tailored to any racial/ethnic minority midlife women [29,30].

The purpose of our study was to identify practical issues in developing and implementing a culturally tailored physical activity promotion program for 2 groups of Asian Americans—Chinese and Korean American midlife women. We targeted midlife women in this study because physical activity in midlife is a significant predictor of better health in later years [33]. Also, midlife women are at increased risk of cardiovascular diseases, type 2 diabetes, obesity, hypertension, and all-cause mortality partially due to high physical inactivity [34-36]. First, we provide background information by concisely summarizing the study’s methods used to identify the issues. Then, we describe and discuss practical issues found during the study process. Finally, we propose implications for future research based on the discussion of these issues.

## Methods

This was a pilot study to determine the effectiveness of a Web-based physical activity promotion program among Chinese American and Korean American midlife women. This study consisted of 2 sections: a usability test and expert review (phase 1) and a preliminary randomized trial (phase 2).

In phase 1, for a usability test, the first 5 Asian American midlife women who participated in previous studies of the research team and who indicated their interests in participating in additional studies were involved in a 1-month-long Web-based forum. The participants were required to come to the forum site, use the program, and post messages with their evaluation of the program within a month. They were asked to evaluate the program on 7 topics, including the general structure of the program, color, designs, and menus of the program, content included in the program, need for technical support and difficulties encountered, links to Internet resources, and other potential issues. Then, we analyzed the users’ posted messages using a content analysis method [37].

The expert review was done by 5 experts in women’s health and Asian American health from our institution. Cultural experts, as well as content experts, were essential for the expert review because the program aimed to be culturally tailored to Asian American midlife women. Then, the experts were provided with the Web address of the program and asked to evaluate the program and send their evaluation by email or by phone within a period of 2 weeks. Then, we analyzed their evaluations using a content analysis method [37].Based on the results from phase 1, the research team made decisions on the directions for program refinement.

For phase 2, we recruited 69 self-reported Chinese or Korean American midlife women. This phase was a randomized repeated-measures pretest-posttest (pretest, post-1 month: time point 1; and post-3 months: time point 2) control group study. The control group did not use the program, but used Internet resources related to Chinese or Korean Americans’ daily life. The intervention group used the program and Internet resources related to Chinese or Korean Americans’ daily life. The Internet resources were those related to daily life concerns and issues of Chinese or Korean Americans (eg, news from Mainland China, Taiwan, or South Korea, Chinese or Korean American businesses in the United States, cooking, and traveling). This phase used multiple instruments, including several questions on background characteristics and health and menopausal status, the Questions on Attitudes toward Physical Activity, Subjective Norm, Perceived Behavioral Control, and Behavioral Intention [38], the Physical Activity Assessment Inventory [39], and the Kaiser Physical Activity Survey [40]. The reliability and validity of all the instruments have been established among Chinese and Korean Americans [41,42].

During the study process, we wrote memos on the practical issues that we identified and our inferences on potential reasons for the issues. We also had weekly group discussions and kept the minutes of the discussions. The memos and minutes were reviewed and analyzed using a content analysis method [37]. No specific software was used for the data analysis due to the small volume of the data. Rather, line-by-line coding, categorization, and theme extraction were used to conduct the content analysis [37]. Each word was a unit of analysis.

## Results

We classified our content analysis findings into 4 idea categories: (1) bilingual translators’ language orientations, (2) cultural sensitivity requirement, (3) low response rate, interest, and retention, and (4) issues in implementation logistics. In the following section, we present and discuss the issues according to the idea categories. Multimedia Appendix 1 also summarizes the practical issues and related implications.

## Discussion

### Practical Issues

#### Bilingual Translators’ Language Orientations

As in the interventions using traditional methods, cultural tailoring in this Web-based intervention required the use of multiple languages. To culturally tailor the Web-based program for Chinese and Korean American midlife women, we used 3 languages for the program: English, Chinese (Mandarin), and Korean. We chose these 3 languages because they are the major languages spoken by Chinese or Korean American midlife women [43].

The program had 3 Web-based components: (1) message boards, (2) educational sessions and one-on-one coaching, and (3) resources. Before starting the study, we prepared the educational sessions and resources in the 3 languages. First, 4 bilingual researchers (2 Chinese-English bilingual researchers and 2 Korean-English bilingual researchers) translated the educational modules and resources into Chinese (traditional Mandarin) and Korean. Then, 4 bilingual researchers checked the accuracy of the translation. We did not use the standard back-translation process [44] for the educational modules and resources because the volume of content was too large.

Bilingualism can lead to some degree of deviance in translated meanings. For example, those from another culture who can speak fluent English do not inevitably have the same cultural beliefs and values of native speakers [44,45]. Also, bilingual translators can bring in some words straight from their second language and use the words and stylistic devices from their second language [44]. Subsequently, the same words can be understood and interpreted differently by different bilingual translators, which can often make scientific translation difficult [46].

Indeed, the main issue that we had in developing the 3 different languages versions of the Web-based program was related to bilingualism [44]. Our issue specifically concerned the equivalence of the bilingual translators’ level of language proficiency in both languages. Although all the translators were identified as bilingual in 2 languages (Chinese and English or Korean and English), some translations tended to be oriented to English sentence structures and wording, while others tended to be oriented to the other languages (Mandarin Chinese or Korean). Thus, the translations could result in awkward sentence structure and wording depending on their language orientations. Thus, for all translations, 2 other bilingual research team members double-checked the translations and wording in the educational modules and forum messages for coaching and support.

#### Cultural Sensitivity Requirement

As in culturally tailored traditional interventions [47,48], cultural sensitivity was essential in developing and implementing the culturally tailored Web-based intervention. First, some cultural issues resulted in difficulties in recruiting research participants. One of the major reasons for the recruitment difficulty was potential participants’ cultural attitudes toward midlife. Many eligible women, particularly those in their 40s, did not want to participate in the study because they did not perceive themselves as midlife women. They felt that midlife women meant those who were much older than them, although they were actually in the midlife age range. Although this attitude can be found in other racial/ethnic groups, it was interesting to find it in Asian American midlife women because traditional Asian cultures gave high respect to elders. With increasing life expectancies and cultural changes in Asian countries [49], Asian (especially Korean) midlife women’s perception of midlife has become quite different from that in traditional Asian cultures. Furthermore, due to negative attitudes toward aging in Asian American cultures, as well as in modern Asian cultures, some women felt offended when we approached them for this study (targeting midlife women), even though this was a Web-based intervention study.

The second hurdle in recruitment was the educational modules and resources related to depressive symptoms that were offered to the intervention group. One of the women dropped out suddenly, so we contacted her to figure out the reason for her dropout. Interestingly, her reason was that she did not want to be involved in any studies with content related to depression. The educational modules were each categorized into 3 topics, such as depression, menopausal symptoms, and physical activity. The reason for introducing the topics of depression and menopause in the program was their relevance to physical activity of midlife women; and one module under each of these topics was specifically designed to inform the relationships of physical activity with depression and menopause. The woman who dropped out may have done so because of the stigma attached to depression in Asian culture [48]. We inferred that her main purpose in joining the study was to promote her physical activities and that she might have perceived the depression topic in the education modules as deviating from her original purpose. Also, due to the cultural stigma attached to depression, she might have misconceived the depression module as a signal to label her as a patient with psychological and psychiatric disorder.

Another culture-related issue was the women’s own perception of their level of physical activity. Because the women perceived any activities as physical activities, they thought they were adequately doing physical activities through their daily life. For example, they even perceived their breathing as physical activities. Indeed, a previous study by Im and Choe found that Korean American midlife women’s definition of physical activity was broad; the women thought only death was physical inactivity, while all other activities could be physical activities [50]. This cultural definition of physical activity was an inhibitor in promoting the women’s physical activity in the study.

Cultural sensitivity was also required in approaching the informal and formal gatekeepers of the Web-based communities for Asian Americans. As a recruitment strategy, we targeted Asian churches across the United States. A research assistant sent emails to the churches’ pastors to ask for their help by announcing the study to their Web-based communities and congregations. However, a problem arose when the research assistant used only English when contacting the churches. We received a response from 1 of the pastors, who gave us advice and tips for effective communication with Asian churches whose services were delivered in Asian languages. To increase the possibility of recruiting church members to the study, he recommended the use of Asian languages (eg, Chinese Mandarin or Korean) in emails; otherwise, pastors would be more likely to ignore or delete the emails, considering them to be advertisement or spam emails. Also, receiving just an email would not make pastors want to help the research. Instead, as the pastor suggested, we needed to call or visit the churches in person and explain the study before asking for help.

A plausible reason for the gatekeepers’ high rate of responses to culturally matched research assistants using their original languages is that the study announcement and communication in their first languages could resonate highly with Asian Americans. Also, collectivism is the moral stance in many Asian cultures, including Chinese and Korean [48]. Asian Americans often give special attention to languages and activities specific to their cultural group. Furthermore, using Asian languages in communication increased potential participants’ sense of belonging, thus increasing the willingness to participate in the study. In addition to their interest in culture-specific activities, Asian Americans also showed special interests in researchers with the same cultural background. For example, one participant in the Chinese group expressed her and her social group’s willingness to support projects conducted by researchers from the same cultural group.

#### Low Response Rate, Interest, and Retention

Web-based recruitment is supposed to be easier than recruitment through traditional methods (eg, mail or phone announcement) due to speedy and flexible communication [51]. However, in this study, recruitment and retention were challenging. Studies have reported high dropout rates in Web-based interventions because the participants could disappear from the website without any difficulty [52]. Indeed, in this study, the dropout rate by the post-3-month survey was 30.43%.

We suspected that the high participation burden for the intervention group (given the amount of participation reimbursement) with a long study period and many requirements from the Web-based program contributed to participants’ withdrawal. The intervention group joined the weekly Web-based forums and completed 3 questionnaires until the end of the study. Also, the intervention group was asked to review 3–4 educational modules and leave questions or thoughts on each module every week. It took approximately one and a half months to cover the entire educational modules. After the first round of the Web-based forum ended, we repeated the same Web-based forum one more time for the intervention group until we invited the participants to fill out the post-3-month survey. One participant who officially withdrew from the study noted the time constraints because she had a full-time job and experienced difficulties setting aside enough time to fulfill the study requirements. Similarly, another participant stopped responding to the research team after being informed of the total number of required educational modules and every week’s commitment to the forum. Others who expressed their interests in the study asked whether a US $30 gift certificate would be offered each time they completed the questionnaire; after hearing that US $30 was the total amount of reimbursement, they decided not to join the study. Therefore, we incorporated additional motivation strategies to prevent further dropouts, such as weekly reminders by emails, a random draw of a US $50 gift card at the completion of the post-1-month survey, and a US $100 gift card at the completion of the post-3-month survey.

The response rate to individual coaching and support by email was also low. A plausible reason is that the intervention group was already overwhelmed by several responsibilities to participate in the forum and did not want to be involved in additional individual coaching or support. Social desirability bias [53] might have affected their participation. Through the individual coaching and support, an interventionist helped the participants set their own goals to promote physical activity, checked their progress each week, provided emotional support, and discussed barriers preventing the participants from increasing their level of physical activity. The participants could feel pressured every time the interventionist assessed how far they had come closer to the goals or why they had not achieved their goals. Participants might have thought they were being judged by the interventionist and blamed for not doing their best. Subsequently, feeling pressured that they should become a good participant complying with the study requirements might have made them not want to participate in the individual coaching and support at all. Also, participants’ characteristics might have influenced the individual coaching and support process. This study limited participation to relatively healthy people who were online by screening out those with current and past medical conditions and even with a family history of cardiovascular diseases. Thus, the healthy participants might have not been highly motivated to increase their physical activity through the individual coaching and support.

#### Logistic Issues in Web-Based Implementation

We had several logistic issues in implementing the Web-based intervention. First, we anticipated that participants would be successfully enrolled into the study within a predetermined enrollment period. In reality, however, their entry points into the study were different and sporadic, which made it difficult to streamline the intervention schedule. Given the limited study period as a pilot study, it was unrealistic just to wait until the intervention group was filled with 25 participants. Thus, we grouped the participants by their enrollment time (those enrolled at similar time points were grouped into a cohort) and then started the intervention until we recruited another cohort. However, even within the same cohort, the starting point of the intervention varied among the participants.

A second issue in implementing the Web-based program was timing, although the Web-based program could be accessed at any time without any geographical restrictions. Because of delays in many administrative aspects of the study, we started the data collection in November. Around the American Thanksgiving holidays, it was difficult to recruit and retain experts for the expert review. Also, it was difficult to recruit and retain Asian American midlife women for the usability test. Several email reminders were needed to get their feedback on the program. Then, because of Christmas holidays, we needed to stop data collection in December. In January, we began to recruit Asian American midlife women through Internet communities. It was still difficult to get responses from the webmasters and Web owners at the beginning of January. Also, there were unexpected Asian holidays (eg, Lunar New Year’s Day) that we did not originally consider in the study implementation, but that turned out to be important to consider during the implementation process.

This study adopted an interactive Web-based platform that was similar to the Facebook platform in order to encourage active discussion and social networking among the participants. Our study website, as in Facebook, allowed the latest postings to appear at the top of the message board. Through several rounds of the forum, we learned that the Facebook-type platform was not working well for the Web-based intervention, where participants’ entry points into the intervention had a wide range even within a single intervention cohort. Specifically, old postings on the message board continued to be pushed down and quickly disappeared because the first page could accommodate only a limited volume of postings. Under this condition, it was very difficult to build up discussions and accumulate comments for each question, especially when multiple cohorts engaged in separate forums with different topics every week. Thus, the interventionist needed to keep posting the questions for newer cohorts on the forum site, although the same questions had already been asked in the past for older cohorts.

### Suggestions for Future Research

Based on the findings, we propose the following suggestions for future research using a full-scale intervention. First, for effective recruitment, it would be critical to assess the characteristics of recruitment sites, either Web-based or offline. In addition, we highly recommend a combined use of Web-based and offline recruitment methods because recruitment only through the Internet may not work anymore due to changes in dynamics (eg, an increasing number of Internet frauds) [54].

Second, as in traditional culturally tailored interventions, bilingual translators with adequate language proficiency in both languages are essential. As our findings indicated, bilingual translators frequently have unbalanced language skills between the 2 languages; they may be more proficient in one language than the other. Thus, having at least one bilingual translator with adequate proficiency in each language is important to ensure the adequacy of translation.

Third, as in traditional culturally tailored interventions, cultural attitudes toward several major concepts and topics related to the study need to be carefully examined. As this study indicated, cultural sensitivity was essential to approach the study population because of several unexpected issues. Usually, Web-based interventions are regarded as stigma-free because of non-face-to-face interactions with research participants [29,31,55]. Thus, many researchers have suggested the use of Web-based interventions for underserved populations with stigmatized conditions and assumed that the participants would not care about their stigmatized condition in Web-based interactions [29,31,55]. However, we found that participants were hesitant to participate in the study because of the cultural stigma attached to several different topics (eg, midlife, depression) that we never expected to cause stigma to the study population in a Web-based environment.

Fourth, more carefully planned motivation strategies need to be adopted. As discussed above, researchers’ perceived adequacy of motivational strategies could be quite different from those of the research participants. The amount of participation reimbursement needs to be carefully set after consulting with some potential participants. In our study, we thought that US $30 would be adequate to motivate participation in the study without any ethical concern (eg, the potential of exploiting low-income persons), but the participants thought that US $30 was too low for their participation.

Fifth, potential issues related to logistics in implementing Web-based interventions need to be carefully considered in the planning stage. Timing and technology-related issues have frequently been reported as disadvantages of Web-based studies in the literature [53-57]. Because of the longitudinal nature of our study, timing was much more important. Once the intervention started, there was no way to stop it while maintaining its continuity despite the upcoming holidays. Thus, timing would be much more important in longitudinal interventions than in one-time interventions. Thus, before starting an intervention, it would be essential for researchers to check culture-specific holidays, as well as national holidays.

### Conclusions

We identified 4 practical issues in developing and implementing a culturally competent Web-based physical activity promotion program for 2 groups of Asian American midlife women (Chinese American and Korean American). The equivalence of bilingual translators’ level of language proficiency in both languages was an issue. Although this was a Web-based intervention study, we also identified several issues related to cultural sensitivity in the use of specific terms and the content of the intervention. We also found low response rates, a low level of interest in the study, and low retention rates despite the use of multiple strategies to motivate the participants. Finally, we found several unexpected issues in implementation logistics (eg, the screen display format).

Based on the findings, we suggest several strategies to overcome these issues; that is, using both Web-based and offline recruitment methods, including bilingual translators with adequate language proficiency in both languages, carefully considering cultural attitudes toward several major concepts and topics related to the study, and carefully planning motivation strategies and implementation logistics. Yet the findings and suggestions need to be carefully interpreted and adopted because of several limitations of the study (eg, recruitment and subject bias, small sample size, and the short timeline).

## Appendix

Multimedia Appendix 1
