# Supplementary material for: Practical Issues in Developing a Culturally Tailored Physical Activity Promotion Program for Chinese and Korean American Midlife Women: A Pilot Study
Source: J Med Internet Res. 2016 Nov 21;18(11):e303. doi: 10.2196/jmir.6454 (PMC5138450; doi:10.2196/jmir.6454)
Supplement: Multimedia Appendix 1 [file jmir_v18i11e303_app1.pdf]

Table 1. A summary of the practical issues and related implications.

Practical Issues	Implications
• The equivalence of the bilingual translators’ level of language proficiency in both languages (language orientations).	• The use of bilingual translators with adequate language proficiency in both languages.<br>• The use of at least one bilingual translator with adequate proficiency in each language.
• Cultural sensitivity issues related to the topics and terms<br>o The use of “midlife.”<br>o Cultural stigma attached to depression.<br>o Cultural different meaning of physical activity.<br>o Cultural matching required.	• Careful considerations on cultural attitudes toward several major concepts and topics related to the study.
• Low responses, interests, and retention<br>o High participant burden (a long study period & multiple requirements).<br>o Low responses due to possible social desirability bias and healthy individuals’ low motivation to increase physical activity.	• Assessment of the characteristics of recruitment sites, either online or offline.<br>• The use of more carefully planned motivation strategies<br>• The combined use of online and offline recruitment methods.
• Logistic Issues in Implementation<br>o Different entry points of the participants.<br>o Timing of the study (e.g., Thanksgiving and Christmas	• Careful considerations on potential issues related to logistics in implementing Web-based interventions.<br>• Careful considerations on timing and technology-related issues in planning

	holidays, Asian holidays).	Web-based interventions.
o	Format issues in online forums.	
